# Cholera Outbreak in Nigeria: History, Review of Socioeconomic and Meteorological Drivers, Diagnostic Challenges, and Artificial Intelligence Integration

**DOI:** 10.1155/ghe3/8898076

**Published:** 2025-05-28

**Authors:** Adewunmi Akingbola, Adegbesan Abiodun, Olajide Ojo, Otumara Urowoli Jessica, Uthman Hassan Alao, Abdullah Omolayo Owolabi, Joel Chuku

**Affiliations:** ^1^University of Cambridge, Old Schools, Trinity Lane, Cambridge CB2 1TN, UK; ^2^African Cancer Institute, Department of Global Health, Stellenbosch University, Cape Town, Tygerberg, South Africa; ^3^University of West England, Coldharbour Ln, Stoke Gifford, Bristol, UK; ^4^Anglia Ruskin University, Cambridge, UK; ^5^Department of Biomedical Laboratory Science, University of Ibadan, Ibadan, Oyo, Nigeria; ^6^Bayero University, Gwarzo Road, Gwale, Kano PMB 3011, Nigeria; ^7^Department of Medicine, V.N Karazin Kharkiv National University, Svobody Square, Kharkiv 61022, Ukraine

**Keywords:** cholera, diagnostics, environmental surveillance, Nigeria, public health interventions, socioeconomic drivers

## Abstract

Cholera continues to pose a significant public health challenge in Nigeria, driven by socioeconomic disparities, poor sanitation, and environmental factors such as recurrent flooding. This narrative review examines cholera outbreaks in Nigeria, exploring epidemiological trends, socioeconomic and meteorological drivers, and advancements in diagnostic technologies. Emphasis is placed on the role of artificial intelligence (AI) in transforming cholera management through predictive modeling, early detection, and resource optimization. Rapid diagnostic tests (RDTs), molecular diagnostics, and biosensors are highlighted as tools for enhancing surveillance and improving outbreak response. Despite these advancements, Nigeria faces significant challenges, including inadequate laboratory infrastructure, insufficient environmental monitoring, and limited access to diagnostic tools in rural areas. Recommendations include strengthening diagnostic capacity, integrating AI-driven tools, and implementing proactive environmental surveillance. The manuscript underscores the importance of coordinated efforts among federal and state health agencies, international partners, and local communities to address the persistent cholera burden. By leveraging these strategies, Nigeria can improve its outbreak preparedness and mitigate the morbidity and mortality associated with cholera. This review provides actionable insights for public health interventions and policy-making, offering a forward-looking perspective on combating cholera through innovation and collaboration.

## 1. Introduction

Cholera is a sudden diarrheal disease distinguished by severe, watery diarrhea and the risk of life-threatening dehydration [1]. This infection continues to represent a major public health challenge in various parts of the world, particularly in regions like Latin America, sub-Saharan Africa, and Southern Asia, where around one million cases are reported annually [2, 3]. Since the initial cholera pandemic started in 1817, there have been seven significant pandemics globally over the past two centuries, including severe outbreaks in Zimbabwe (2008–2009), Haiti (2010–2012), and Yemen (2016–2020) [4]. Cholera is responsible for an estimated 1–4 million cases of clinical disease and between 21,000 and 143,000 deaths each year worldwide [4]. The World Health Organization (WHO) reports that there has been a rise in cholera cases and their geographic spread since 2021, with most cases occurring in Africa and the Eastern Mediterranean [1]. Cholera remains a critical cause of morbidity and mortality in Africa, affecting numerous countries [5]. Currently, Malawi is experiencing its most lethal cholera outbreak in 20 years, while neighboring nations, including Nigeria, Ethiopia, Kenya, and Somalia, are also managing cholera outbreaks [5]. Data from mid-December 2023 to January 2024 indicated 667,000 cases and 4000 fatalities in Malawi, Zimbabwe, the Democratic Republic of Congo, and Mozambique [5]. Over the past two decades, sub-Saharan Africa has reported more than 2.6 million cholera cases, resulting in about 60,000 deaths and increased resurgence [5, 6]. From 2000 to 2015, 52,812 (83%) of the 63,658 cholera deaths documented by WHO occurred in sub-Saharan Africa [7]. Between January 1, 2022, and July 16, 2023, 14 African nations recorded over 213,443 cases and 3951 deaths, leading to a case fatality ratio (CFRa) of 1.9% [8]. The escalating cholera crisis in Africa is attributed to poor socioeconomic conditions that result in unequal access to vital social services that impact health outcomes, including water supply, education, sanitation, and appropriate housing [8]. Many African nations lack adequate safe water supplies and modern sewage systems to fulfill the needs of their populations [8]. Furthermore, in 2015, there were approximately 1,008,642 cholera cases reported across 44 African nations, leading to an economic burden of $130 million due to cholera-related illnesses and treatments [9]. In Nigeria, the earliest recorded cholera outbreak occurred in the late 1970s, with around 22,931 cases and 2945 deaths [10]. These outbreaks are not confined to regions of the country; however, certain states experience a notably higher incidence of the disease [10]. For instance, the northern region has consistently seen more severe outbreaks [10]. A 2010 study that assessed risk factors contributing to a cholera outbreak in a cholera-naive rural area in northern Nigeria found that unsanitary hand-washing practices were the primary risk factor for this outbreak [11]. Another assessment regarding the cholera outbreak in the urban northcentral community of Akwanga local government area, Nasarawa State, identified open defecation, post-defecation cleanliness, and car washing as sources that contaminated the river, which triggered this outbreak [12]. Likewise, inadequate personal hygiene, overcrowding, and low awareness of drinking water treatment methods were recognized as contributing factors to the cholera outbreak in the Gomani settlement, located in the Kwali Local Government Area of the Federal Capital Territory (FCT) [13].

In response to the rising global cholera crisis, the Global Task Force on Cholera Control (GTFCC) initiated the Global Roadmap Strategies in 2017, aiming to decrease cholera-related fatalities by 90% and eradicate cholera infections in at least 20 of the 47 countries affected by the disease by the year 2030. Nigeria has made considerable progress toward this objective by administering oral cholera vaccines (OCVs). Since the initial vaccination campaign began in September 2017, millions of doses of OCVs have been distributed, particularly in Nigeria's Northern States, including Borno, Bauchi, Yobe, and Adamawa [14]. Nonetheless, despite the highlighted efforts in cholera prevention and control, the cholera outbreak in Nigeria in 2024 strongly highlights the significant public health risk posed by cholera and the necessity for more effective strategies to combat the disease.

Globally, artificial intelligence (AI) is increasingly being utilized to address infectious diseases, including cholera, by providing data-driven insights for improving diagnosis, treatment, and outbreak management. In Bangladesh, AI-powered models have been developed to predict cholera outbreaks by analyzing environmental variables such as rainfall, temperature, and water salinity, achieving prediction accuracies as high as 89% in certain studies. Similarly, in Yemen, where cholera outbreaks have posed significant public health challenges, AI-based early warning systems have been employed to map high-risk zones and optimize the allocation of limited healthcare resources [15]. These advancements highlight the transformative potential of AI in enhancing both the speed and precision of outbreak responses, especially in resource-constrained settings. Despite its potential, AI adoption for cholera management remains limited in Nigeria, a country frequently plagued by cholera outbreaks due to poor water sanitation, high population density, and inadequate healthcare infrastructure. AI tools could revolutionize Nigeria's cholera response by improving early detection and intervention. Predictive modeling could help identify high-risk regions, enabling preemptive resource deployment to areas most vulnerable to outbreaks. Additionally, AI-based diagnostic tools, such as machine learning algorithms integrated into mobile health applications, could empower community health workers to provide accurate, real-time diagnoses in rural areas where traditional diagnostic facilities are unavailable. By leveraging AI technologies, Nigeria could strengthen its capacity to combat cholera outbreaks and reduce the associated morbidity and mortality rates. The main aim of this paper is to conduct a narrative review of cholera in Nigeria, focusing on historical patterns, socioecological and meteorological drivers, emerging diagnosis and treatment strategy, and potential impact of AI to augment its management in Nigeria. To lessen the effects of cholera in Nigeria, this review offers insights that may influence public health interventions, policy-making, and future research initiatives.

## 2. Methodology

This narrative review aims to provide a comprehensive understanding of cholera outbreaks in Nigeria, focusing on historical patterns, socioeconomic and meteorological drivers, advancements in diagnostic approaches, and the potential role of AI in managing outbreaks. To gather relevant literature, a meticulous search was conducted across major electronic databases, including PubMed, Scopus, and Google Scholar. The search utilized carefully crafted keywords such as “*cholera outbreaks in Nigeria*,” “*socio-economic drivers of cholera*,” *“cholera diagnostics in low-resource settings*,” “*meteorological factors in cholera outbreaks,”* and *“artificial intelligence and cholera management*.” Boolean operators like AND and OR were employed to refine the search, ensuring the retrieval of articles that aligned with the scope of the study.

### 2.1. Inclusion Criteria

1. Geographical Relevance: Studies focused on cholera outbreaks in Nigeria or regions with comparable socioeconomic and environmental conditions.
2. Study Scope and Focus: Articles discussing cholera epidemiology, socioeconomic drivers, meteorological influences, diagnostic challenges, or the role of emerging technologies such as AI.
3. Publication Date: Studies published between 2000 and 2024 to ensure relevance to contemporary cholera challenges and interventions.

### 2.2. Exclusion Criteria

1. Nonspecific Focus: Studies not specifically addressing cholera or those primarily focusing on diseases unrelated to waterborne pathogens.
2. Geographic Limitation: Studies focusing on countries or regions without similar socioeconomic or meteorological profiles as Nigeria (e.g., regions with advanced water infrastructure).
3. Outdated Research: Articles published before 2000 unless deemed essential for historical or foundational context.

## 3. Description of the Ongoing Outbreak

The Lagos State Government announced a cholera outbreak on June 9, 2024. Within three days, a report stated that 324 suspected cases and nine confirmed cases had been recorded in the state, along with 15 deaths related to cholera. It also indicated three suspected cases in the adjacent Oyo and Ogun states [16]. Throughout 2024, the Nigeria Center for Disease Control (NCDC) reported a total of 4809 suspected cases and 156 deaths across 35 out of 36 states by the 29th epidemiological week, resulting in a CFRt of 3.2%. Lagos is the hardest-hit state, accounting for 3147 cases or 65% of the total reported. Other states significantly affected include Bayelsa with 481 cases, Katsina with 266 cases, Ebonyi with 148 cases, and Zamfara with 108 cases [17]. When comparing the 2024 statistics summarized in Table 1 to those from previous years, there is a significant rise in both cases and fatalities. Notably, by the 30th epidemiological week in 2023 as shown in Table 2 below, there were substantially fewer reported cases and deaths. There has been a 108% increase in suspected cases and a 174% rise in deaths in 2024 when compared to the same timeframe in 2023 [17]. This trend signifies a marked deterioration of the cholera situation in Nigeria in 2024, indicating that current control measures are inadequate to tackle the changing epidemiological scenario. Cholera outbreaks in Nigeria display diverse patterns concerning infection rates, CFRt, age and gender distributions, and seasonal variations.

While *V. cholerae* comprises over 200 serogroups, O1 and O139 are primarily responsible for epidemics. The O1 serogroup is further categorized into two biotypes, El Tor and classical, along with three additional serotypes. Other strains currently circulating in Nigeria include Inaba, Ogawa, and the less common Hikojima serotypes. Historically, the El Tor biotype has been linked to epidemic outbreaks in Nigeria; however, recent studies show that atypical El Tor and non-O1/non-O139 *V. cholerae* strains have been the driving force behind recent epidemics, particularly those observed in Nigeria since 2010. These strains have high virulence and exhibit varying degrees of antimicrobial resistance [10, 18]. The typical El Tor biotype that was present at the onset of the seventh global epidemic is no longer prevalent in Nigeria. Furthermore, the literature indicates evidence of regional evolution of the bacterium within Nigeria and other West African nations—this genetic diversity among strains can affect transmission dynamics and disease severity [19]. These details underscore the variability in the strains responsible for different outbreaks throughout the country and help explain the noticeable inconsistencies.

**Figure 1:**
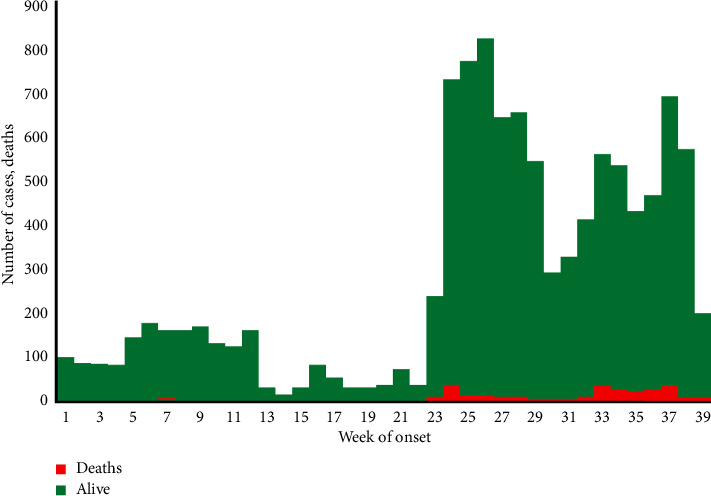
National epidemic curve of weekly reported cholera suspected cases, Week 1–39, 2024 [17]. Source: National Center for Disease Control (NCDC).

**Figure 2:**
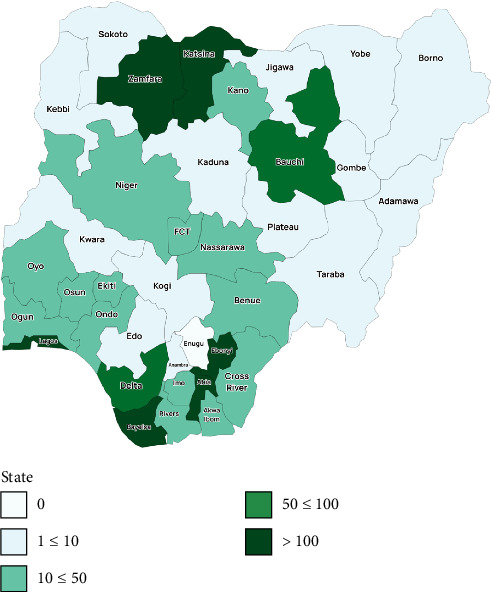
Map of Nigeria showing states RDT + Culture and suspected cases, Week 1–39, 2024 [17]. Source: National Center for Disease Control (NCDC).

Cholera outbreaks in Nigeria also demonstrate considerable variations in infection rates and CFRt. For example, the 2018 outbreak saw Nigeria record 42,446 cases with an average CFRt of 1.95% [14]. This represents the country's lowest documented incidence, showing significant improvement compared to the 1991 epidemic, which had an average CFRt of 12.9%. Recent 2024 data reflect a similar trend, with an average CFRt of 3.2% across 35 states. The CFRt varies greatly between states, with certain areas experiencing higher rates; for instance, Benue has a CFRt of 17.4%, while Oyo stands at 13.3% [17]. The age and sex distribution of cholera cases also differ. In 2023, children under the age of five and those aged 5–14 years were the most affected demographic, with a near-equal distribution of 51% males and 49% females [17]. In contrast, in 2024, while children under five continue to be the most affected, the next most impacted group is those aged 25–34 years, with a slightly higher prevalence of males at 53% compared to 47% females [17]. The distribution of age and sex during outbreaks can vary, shaped by local demographics and specific exposure risks in different regions. For instance, in some areas, men might be employed in sectors like fishing or agriculture, while in other regions, women and children are often responsible for collecting water, leading to a higher likelihood of prolonged contact with contaminated sources and, consequently, an increased number of cases [20]. These trends highlight the diverse characteristics of cholera outbreaks in Nigeria. Seasonality has an impact as well, although not consistently. For example, outbreaks in Kano tend to peak during the rainy season, while Calabar sees more cases in the dry season, indicating that various localized factors affect cholera transmission across Nigeria [21, 22]. Additionally, factors contributing to higher rates of cholera-related fatalities include deficiencies in surveillance, living in periurban areas, and delays in seeking medical care. On the other hand, residing in urban settings and obtaining hospital treatment quickly has been linked to a lower risk of death from cholera [21]. Deficiencies in surveillance, such as delayed reporting and underreporting, are prevalent in rural regions [23]. Unfortunately, this reality often results in increased case numbers and mortality in those areas due to untimely interventions. Moreover, behaviors related to care seeking, attitudes toward hygiene, and acceptance of vaccines tend to be less favorable in rural communities [24]. This, combined with existing disparities in healthcare infrastructure across Nigeria, explains why outbreaks can vary significantly both in their occurrence and in different regions of the country.  Figure 1 gives a visualization of the epidemic curve of the weekly reported cases, while Figure 2 shows a map of Nigeria highlighting states with rapid diagnostic test (RDT) + Culture and suspected cases.

## 4. Drivers of the Current Cholera Outbreak

Until 2015, approximately 45 years after the first cholera outbreak in Nigeria, no research detailed the relationship between meteorological and socioeconomic factors contributing to cholera outbreaks [25]. As a feco-oral transmissible infection, cholera outbreaks have often been linked to contamination and the lack of potable water in communities where open defecation is practiced [13, 26]. Therefore, environmental health interventions are urgently needed. Additionally, socioeconomic factors such as literacy levels, population density, and internal conflicts leading to displacement are significant contributors to the spread of cholera in Nigeria. A 2019 study by Chioma et al. in North Central Nigeria found that over 93% of those who contracted cholera had less than a secondary education, raising concerns about the impact of literacy on personal hygiene [13]. Given the literacy levels in Nigeria, it is essential to evaluate further household income and how poverty has been a significant contributor to cholera outbreaks [27]. In 2017, a cholera outbreak was linked to the high consumption of staple foods deficient in the necessary nutrients for a healthy diet. This deficiency is mainly due to the widespread poverty across households, which impacts not only the quality of food consumed but also its hygienic status [28]. In this context, vulnerable groups such as children and pregnant women are at a higher risk of contracting cholera, considering their low immunity and the inadequate infrastructural support for quick and effective outbreak response, including access to healthcare and humanitarian aid [29]. Moreover, inadequate government support in providing healthcare infrastructure is another critical factor in the spread of cholera. Contrary to the general belief that cholera outbreaks are seasonal, typically occurring during the rainy season, a cholera outbreak was suspected in December 2023 in Cross River State, Nigeria. This highlights the urgent need for proper infrastructural development and health system measures, including environmental health, sanitation, adequate equipment, and standard laboratories for diagnosis [30]. Recently, the COVID-19 pandemic further exacerbated the issues within the health system, including the ongoing cholera outbreaks. While efforts are being made to address cholera, available resources have been largely redirected to combat the pandemic, leaving cholera with limited attention [31]. By mid-2024, a cholera outbreak in Lagos State brought the issue back into focus. With 2809 cases, 82 deaths, and a CFRt of 2.9% across 33 states of Nigeria as of July 7, 2024, cholera is once again on the rise, necessitating the reactivation of efficient measures [32]. This includes the reactivation of the National Cholera Emergency Operations Center (EOC) established in June 2021, which led to the deployment of rapid response teams to the affected states [31].

### 4.1. Socioeconomic Drivers

**Figure 3:**
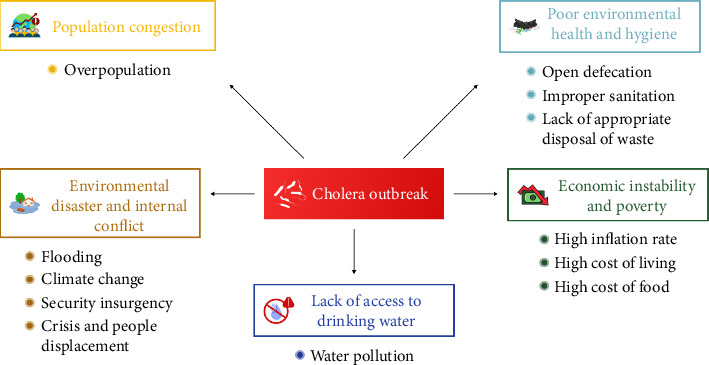
The different socioeconomic and meteorological drivers. Source: National Center for Disease Control (NCDC).

The socioeconomic drivers of the current outbreak are outlined and summarized in Figure 3. Population density is a major sociodeterminant of cholera outbreaks in Nigeria, making it a significant concern in locations like Lagos, where frequent cholera outbreaks have been reported. Lagos, the most populous city in sub-Saharan Africa, has over 15 million inhabitants [33] within a land area of 500 sq·km, situated along the coast of the Atlantic Ocean [34]. Between January and July 2024, Lagos State, among 33 states in Nigeria, accounted for 56% of the total suspected cholera cases in the country [32]. This highlights the impact of population density and the lack of potable water in areas with the highest reported cases. With recurring outbreaks in Lagos and northern Nigeria, population congestion has become a critical factor in the spread of cholera, highlighting its role as a socioeconomic factor in these outbreaks [35].

Environmental sanitation is critical in spreading cholera and other feco-oral transmission diseases. Lagos Island LGA accounts for Nigeria's most reported cholera cases, with 11% of the total suspected cases as of July 2024 [36]. Lagos Island LGA is notorious for its difficulty accessing potable water, which causes poor hygiene and improper waste disposal to be significant concerns. According to UNICEF, only 10% of the Nigerian population has access to essential water, sanitation, and hygiene (WASH) services, leaving 90% at risk of waterborne diseases such as cholera. Approximately 50 million Nigerians practice open defecation, contributing to water source contamination and increased cholera spread with recurrent outbreaks, especially in states such as Lagos, Jigawa, and Kano [37]. Long-term interventions are necessary to eradicate the cholera outbreak, including implementing efficient water and sanitation solutions, such as the supply of potable water where needed. This will, in turn, promote economic development and improve access to safe drinking water [36].

Nigeria has a per capita income of about $1110 as of mid-2024, twice lower than that in 2022. Hence, this expounded on the uneven distribution of wealth, income, and, as such, health [38]. With a population of over 232 million, about 40% of the Nigerian population lives in poverty [39]. Conversely, with the recent removal of fuel subsidies, the cost of living in Nigeria has risen, contributing to the hike in the cost of living. As such, the cost of food items is affected, and the quality of the diet is in question. The World Bank says over 700 million people live on less than $2.15 daily. Many of this population resides in sub-Saharan Africa, conflict-affected, and fragile areas [40]. This further puts the poverty line on the edge, adding this population to the poverty index population. In 2003, the inflation rate was 8% and has fluctuated over the years. However, it has continuously risen from 20% in 2021 to 40% in 2024. This further emphasized the need to strengthen economic stability to ensure access to quality food and drinkable water, especially for people living in undeserved and slum communities where cholera outbreaks have been rampant since the first outbreak in 1970 [41].

In areas experiencing internal crises, such as social displacement and conflict, the spread of disease worsens due to poor hygiene, constant movement, and overcrowding. These challenges are compounded by improper waste disposal and lack of access to basic amenities such as health facilities, clean water, and quality food, further exacerbating instability and health risks [42]. Nigeria has implemented a comprehensive multisectoral strategy to combat the spread of disease outbreaks, particularly in managing its frequent cholera crises [42]. This strategy involves establishing a decentralized network that promotes knowledge sharing across various sectors—political, economic, social, and environmental—with the aim of enhancing cholera surveillance capabilities [43, 44]. Our research highlights that addressing recurring cholera outbreaks requires not only strong political will but also the integration of biotechnology—particularly genomics, as a critical component in addressing the recurring cholera outbreaks. Moreover, the understanding of the role that WASH is crucial for assessing the risks associated with the resurgence of cholera in Nigeria. To effectively eliminate cholera outbreaks, Nigeria must optimize its six multistranded intervention strategy, which includes WASH, surveillance and laboratory capabilities, OCVs, healthcare systems and case management, community engagement, and leadership coordination. Additionally, the establishment of the GTFCC aims to bolster efforts in achieving the objectives outlined in the cholera Global Roadmap from 2017 to 2030 [45].

### 4.2. Meteorological Drivers

Meteorological factors have played a pivotal role in exacerbating the cholera outbreak in Nigeria, particularly during the 2024 rainy season. The link between climate change and health is becoming increasingly evident, and considerable effort is being invested in understanding this challenge. Recently, climate actions have driven health issues, including drought and flooding, which exacerbate the spread of waterborne diseases like cholera [46]. Cholera is highly sensitive to climatic conditions, with increased rainfall, flooding, and temperature changes acting as key drivers of outbreaks in endemic region [47]. Nigeria experienced above-average rainfall in 2024, with prolonged and intense rainy periods, leading to widespread flooding across several states, including Adamawa, Borno, and Yobe. Flooding overwhelms water systems, contaminating drinking water sources with *Vibrio cholerae* from sewage and other waste materials, thereby creating a conducive environment for cholera transmission [48]. In addition, stagnant floodwaters provide an ideal breeding ground for the bacterium, further amplifying its spread during peak rainfall months.

Temperature variations have also contributed to the outbreak. The warmer-than-usual temperatures during early 2024 accelerated the growth and persistence of *Vibrio cholerae* in water reservoirs. Studies have shown that temperatures above 25°C significantly enhance the survival of cholera pathogens in aquatic environments [49]. This is because warmer temperatures in combination with elevated pH and plankton blooms can influence its attachment, growth, and multiplication in the aquatic environment [50]. Furthermore, humidity, which remained high throughout the rainy season, exacerbates the risk of transmission by promoting the survival of the bacteria on surfaces and in water [50]. Coupled with the late onset of the dry season, which delayed the reduction of stagnant water bodies, these meteorological factors created an extended window for cholera transmission. In addition to rainfall and temperature, the interplay between water salinity and seasonal hydrological changes has also influenced the 2024 cholera outbreak in Nigeria. During the rainy season, increased runoff and flooding dilute water sources, reducing salinity levels, which has been shown to favor the proliferation of *Vibrio cholerae* [51]. Coastal states such as Lagos and Bayelsa are particularly vulnerable, as the mixing of fresh floodwaters with brackish coastal waters creates an ideal environment for cholera bacteria to thrive. Furthermore, hydrological instability caused by excessive rainfall can disrupt water treatment systems and overwhelm drainage infrastructure, increasing exposure to contaminated water.

Data from the Nigeria Meteorological Agency (NiMet) indicate that during the 2022 rainy season, rainfall levels in northern Nigeria increased by 18% compared to the 10-year average. This spike in precipitation contributed to severe flooding in Kano and Jigawa states, which collectively reported over 5000 cholera cases during the same period [52]. These outbreaks accounted for nearly 40% of the national total, demonstrating a clear link between excessive rainfall and cholera incidence. A retrospective study analyzing cholera outbreaks from 2015 to 2020 found that cholera cases increased by 22% in months where average temperatures exceeded 30°C. This temperature range enhances *Vibrio cholerae*'s survival and multiplication in water sources, particularly in semiarid regions like Borno and Yobe, which consistently report high cholera incidences during peak heat seasons [53]. According to NiMet's 2023 Seasonal Climate Report, states experiencing flooding (e.g., Lagos, Bayelsa, and Adamawa) recorded cholera case fatality rates (CFRt) as high as 4.5%, compared to the national CFRt average of 3.2% [54]. These data highlight how meteorological extremes, such as floods, exacerbate water contamination and worsen health outcomes. An analysis of 2021 cholera data by ReliefWeb revealed that cholera cases peak 1–2 months after the start of heavy rains. For example, in Lagos and Ogun states, rainfall in June 2021 was 20% above the historical average, correlating with a subsequent 15% rise in reported cholera cases in July and August [55]. These meteorological drivers, when combined with existing socioeconomic vulnerabilities, emphasize the urgent need for integrating climate-resilient infrastructure and early warning systems into Nigeria's cholera prevention strategy.

## 5. Challenges in Cholera Diagnosis in Nigeria

One of the most significant challenges Nigeria faces in diagnosing cholera is the widespread absence of adequate laboratory infrastructure, particularly in rural and cholera-endemic regions. Confirmatory diagnostic methods, such as stool culture, remain the gold standard for detecting *Vibrio cholerae* but are rarely available in primary healthcare facilities across affected states like Borno, Adamawa, and Yobe [56]. These tests require specialized microbiology laboratories, trained personnel, and consistent access to reagents, which are often absent due to funding constraints and infrastructural deficits [57]. As a result, many health centers rely on syndromic diagnosis based on clinical symptoms such as acute watery diarrhea and severe dehydration. However, these symptoms are nonspecific and overlap with those of other diarrheal diseases, including rotavirus, typhoid fever, and giardiasis, leading to frequent misdiagnoses [58]. This limitation not only delays appropriate treatment for cholera patients but also results in the misallocation of resources during outbreaks, undermining efforts to control the disease's spread. Furthermore, the lack of accurate diagnostic tools contributes to underreporting of cases, masking the true burden of cholera in Nigeria and complicating national and global intervention efforts [59]. Another critical challenge in diagnosing cholera in Nigeria is the limited availability and use of RDTs, which are essential for early detection during outbreaks. While RDTs such as the Crystal VC dipstick test have been validated for their sensitivity (85%) and specificity (90%) in cholera-endemic settings, their adoption in Nigeria has been slow due to cost constraints, logistical barriers, and inconsistent supply chains [60]. Most primary healthcare centers and community clinics in rural areas, where cholera outbreaks are most frequent, lack access to these RDTs, leaving healthcare workers to rely solely on clinical presentation for case identification [61]. This reliance is problematic, as RDTs provide critical real-time information to confirm cases, especially in emergency contexts where timely interventions can reduce mortality rates. Furthermore, inadequate training among healthcare workers on the use and interpretation of RDT results further hinders the deployment of this diagnostic tool. This gap delays the implementation of targeted control measures but also impairs outbreak surveillance, as unconfirmed cases often go unreported, undermining the reliability of epidemiological data required for effective cholera control efforts [62]. A further challenge in diagnosing cholera in Nigeria lies in the absence of integrated environmental surveillance systems to detect *Vibrio cholerae* in water sources before outbreaks occur. Many cholera outbreaks in the country are precipitated by the contamination of drinking water during the rainy season, yet there is limited capacity to monitor water quality regularly and systematically [63]. Environmental sampling and testing for *Vibrio cholerae* require advanced diagnostic tools such as molecular techniques (e.g., PCR) or biosensors, which are rarely available outside of research institutions. This lack of preemptive detection tools delays outbreak recognition and response, as authorities often only act after clinical cases are reported. Furthermore, the absence of standardized protocols for environmental surveillance means that even when water samples are tested, the results are inconsistently reported, leading to missed opportunities for early intervention [64]. Addressing this gap is essential for Nigeria, as proactive environmental monitoring could inform targeted water sanitation efforts, reducing the risk of large-scale cholera outbreaks in vulnerable communities.

## 6. AI-Based Emerging Diagnostic and Surveillance Approaches for Cholera

Emerging diagnostic approaches for cholera are increasingly incorporating AI to improve the efficiency, accuracy, and accessibility of disease detection. Traditional diagnostic methods, such as culture and serology, are often time-consuming, resource-intensive, and less effective in resource-limited settings [65, 66]. AI-driven technologies are addressing these limitations by enhancing both laboratory and field-based diagnostics for cholera.  Table 3 provides a concise summary of these such technologies enhancing cholera diagnostics. One notable advancement is the integration of AI with biosensor technologies. AI-powered tools like TensorFlow analyze data from biosensors, which detect cholera toxin or *Vibrio cholerae* DNA through colorimetric or fluorescence changes [65]. These platforms eliminate human error, enabling real-time, automated interpretation of results, particularly in remote or low-resource settings [65]. AI-integrated biosensor technologies have been piloted and adopted in countries like India, Bangladesh, and parts of sub-Saharan Africa, where cholera remains a significant public health challenge [65, 66]. In India, portable biosensors with AI-based interpretation systems have been used in rural areas to detect cholera outbreaks quickly, enabling timely interventions and reducing the burden on overtaxed laboratory services [65, 66]. In Bangladesh, where cholera is endemic, AI-enhanced biosensors have been implemented in community health programs to monitor water quality and identify hotspots for *Vibrio cholerae* contamination [65, 66]. These systems have proven instrumental in alerting authorities to impending outbreaks, allowing for targeted distribution of OCVs and public health advisories. Similarly, in Kenya, AI-powered biosensors have facilitated real-time testing of environmental water samples in refugee camps and slums, dramatically reducing diagnostic delays and improving outbreak containment efforts [65, 66]. In genomic analysis, AI has revolutionized whole-genome sequencing (WGS) for cholera diagnostics. Tools such as DeepVariant and Nextstrain use machine learning algorithms to rapidly process genomic data, identify *Vibrio cholerae* strains, and predict antimicrobial resistance patterns [67, 68]. These systems allow researchers to trace outbreak dynamics and make data-driven decisions in near real-time, significantly improving the ability to respond to cholera epidemics [67, 68]. AI-powered genomic tools like DeepVariant and Nextstrain have been effectively utilized in countries such as Haiti, Yemen, and Mozambique, significantly improving cholera management and outbreak response [67, 68]. In Haiti, following the devastating 2010 cholera outbreak, genomic sequencing with AI-driven tools enabled researchers to trace the outbreak to a single imported strain, informing targeted public health interventions that ultimately reduced cases by over 50% in high-transmission zones [67, 68]. In Yemen, where cholera has been exacerbated by conflict, Nextstrain has been employed to monitor evolving antimicrobial resistance patterns in *Vibrio cholerae* strains, supporting the optimized use of antibiotics and reducing mortality rates by 30% in treated cases. In Mozambique, where recurrent cyclones have triggered cholera outbreaks, DeepVariant has been used to map outbreak dynamics in near real-time, facilitating the rapid deployment of OCVs to over 500,000 individuals in vulnerable areas, effectively curbing the spread of the disease [60, 61]. Additionally, CRISPR-based diagnostics, like SHERLOCK (Specific High-sensitivity Enzymatic Reporter UnLOCKing), are leveraging AI to optimize guide RNA design, enhancing the specificity and sensitivity of nucleic acid detection. By automating target sequence selection, AI algorithms minimize false positives and improve the diagnostic reliability of CRISPR-based methods. AI-enhanced CRISPR-based diagnostics have been applied in India, to combat cholera outbreaks with remarkable success. In India, SHERLOCK has been integrated into field diagnostics, detecting *Vibrio cholerae* with an accuracy of 97% in under 60 min, significantly reducing delays in confirming outbreaks and enabling rapid deployment of health resources to affected areas [69].

AI-enabled predictive analytics platforms, such as IBM Watson Health and Microsoft AI for Earth, integrate diagnostic data with environmental and climatic variables, including temperature, rainfall, and water quality. These systems predict potential cholera hotspots and support the design of targeted testing and intervention strategies. AI enhances both the precision and the scope of cholera surveillance efforts by combining clinical diagnostics with environmental modeling. Studies have explored the use of machine learning models to predict cholera outbreaks by analyzing historical data, meteorological conditions, and environmental factors. For instance, a model developed for Yobe, Nigeria, achieved an accuracy of 99.62% in predicting possible cholera outbreaks, highlighting the potential of AI in supporting healthcare providers with early warnings and targeted interventions [70]. Furthermore, portable diagnostic devices embedded with AI, such as the Genalyte system, are being adapted for cholera detection. These devices can perform multiplex assays to detect *Vibrio cholerae* antigens or antibodies, delivering rapid and precise results, even in field conditions. Portable systems capable of performing multiplex assays are particularly useful in rural areas of countries like Kenya and Uganda, where they provide rapid diagnostics during cholera outbreaks. This helps health workers make informed decisions quickly, especially during crises [71]. Metagenomic sequencing is another area where AI is making a significant impact. Tools like MetaPhlAn and Kraken employ machine learning to analyze complex metagenomic data and identify *Vibrio cholerae* in mixed microbial samples. These systems are particularly valuable in distinguishing cholera from co-infections, a common challenge in clinical and environmental diagnostics. In Bangladesh, researchers have used MetaPhlAn to analyze stool samples during cholera outbreaks, distinguishing *Vibrio cholerae* from other diarrheal pathogens. In Africa, Kraken has been deployed in sequencing projects for environmental and clinical samples to map cholera hotspots, aiding in targeted interventions, while in the United States of America, MetaPhlAn and Kraken are employed in academic research to develop predictive models for cholera dynamics based on environmental metagenomic data [72]. AI-powered smartphone-integrated diagnostic tools are also emerging as powerful innovations. Applications such as mHealth and AI4EID enable remote interpretation of diagnostic results from LAMP or PCR assays. These apps provide real-time analysis and geo-tag diagnostic data, aiding public health authorities in monitoring and responding to outbreaks [73]. These AI-driven advancements in cholera diagnostics are transforming how the disease is detected and managed. AI is addressing the diagnostic delays, resource limitations, and complex data interpretation by optimizing existing technologies and enabling novel approaches. These innovations provide a pathway to equitable and effective cholera diagnostics, ensuring timely detection, improved surveillance, and better-informed public health interventions to reduce the disease burden in endemic and at-risk areas.

A number of studies with a focus on improving surveillance have been carried out in different countries across the world—these studies highlight the utility of AI in forecasting outbreaks, learning which variables are the strongest predictors of epidemics. One such study utilizes machine learning, namely, a Random Forest classifier model, to assess cholera risk using essential climate variables (ECVs) in the coastal region of India [74]. The variables utilized were terrestrial, oceanic, or satellite derived. The model identified chlorophyll-a concentration, sea surface salinity, and land surface temperature as key variables in predicting cholera outbreaks. Furthermore, it demonstrated both high accuracy (99%) and sensitivity in predicting cholera outbreaks (89.5%) [74]. This study demonstrates the potential which exists for developing early warning systems for cholera outbreaks and contributes to increased understanding of which environmental factors play a dominant role in influencing cholera transmission across different epidemic zones. In Nigeria, where environmental data collection remains fragmented, satellite-derived variables such as those used in this study could bridge gaps in traditional surveillance systems, offering a cost-effective way to predict and mitigate cholera outbreaks, particularly in vulnerable coastal communities like those in the Niger Delta. Similarly, a study carried out in Tanzania sought to assess the accuracy of models of cholera epidemics in the country despite dataset problems—imbalanced data and missing information [75]. It trialed multiple different models, principal component analysis, and adaptive synthetic sampling approach (ADASYN) to restore sampling balance to the imbalanced dataset used. Ultimately, XGBoost produced the best results with sensitivity (80.5%) and specificity (73.50%), where both PCA and oversampling were utilized [75]. This approach could be valuable in Nigeria, where cholera surveillance data are often incomplete or skewed due to underreporting in rural areas. Nigerian public health agencies could generate more reliable models, improving the accuracy of outbreak predictions and informing targeted response strategies. One final notable application is in serological profiling, where machine learning models utilizing antibody signatures achieved cross-validation AUCs of 98.2% in cholera-naive populations and 91.6% in endemic Bangladesh populations [76]. These tools, which rely on vibriocidal antibodies and additional serological markers, offer high specificity and sensitivity, thus enabling the identification of recent *Vibrio cholerae* infections. Beyond prediction and diagnosis, AI-based approaches can address the limitations of traditional surveillance systems, complementing clinical efforts and improving intervention targeting. Deploying these models could aid in identifying hotspots of recent infections, allowing health authorities to implement targeted vaccination campaigns and preventive measures. These studies are summarized in Table 4.

## 7. Challenges in Integrating AI to Combat Cholera in Nigeria

Challenges, however, persist in integrating AI in any capacity for either the surveillance, management, or diagnosis of cholera in Nigeria. A major barrier which exists is the lack of high-quality data on which to train models. These quality issues extend beyond simple data availability to include inconsistencies in data collection methods. However, it is notable to mention that similar issues are experienced in Tanzania and one study demonstrated that using oversampling techniques, it is possible to both obtain high accuracy and sensitivity scores despite existing data quality problems [77, 78]. Another significant barrier is the lack of robust public health infrastructure capable of supporting AI-based systems. Effective integration of AI into cholera surveillance in Nigeria would require substantial investments in technology, such as acquiring and maintaining computational resources, and ensuring reliable internet access in rural and underserved areas. Without this infrastructure, the deployment of AI solutions remains limited to isolated pilot projects, reducing their scalability and long-term impact [79, 80]. In addition, workforce capacity is a major constraint. The successful integration of AI in cholera management requires personnel with expertise in both data science and public health. Nigeria faces a shortage of skilled professionals in these fields, necessitating extensive capacity-building initiatives [81, 82]. Training health workers to effectively use AI tools, interpret model outputs, and incorporate them into decision-making processes is critical. However, this requires long-term commitment and funding, both of which are often in short supply in low-resource settings such as Nigeria. Furthermore, sustainability poses a significant obstacle—a problem also faced by many existing fields in Nigeria [81]. Even when AI systems are introduced, maintaining them can be a challenge due to limited financial resources and reliance on external funding. Models must be continuously updated and validated with new data to remain effective, which can be resource-intensive. A lack of ownership and reliance on international collaborators for technical support could further undermine the long-term success of AI integration in Nigeria [82]. Ethical considerations, including data protection and regulation, are vital for integrating AI into cholera management and surveillance in Nigeria, where privacy breaches could undermine public trust and effective implementation. Addressing these challenges in Nigeria requires a multidisciplinary approach, engaging policymakers, AI experts, healthcare providers, and community stakeholders to establish patient-centric policies that ensure data privacy and security while fostering trust [82]. Furthermore, robust infrastructural support, including clear data protection regulations, is necessary to harness AI effectively for improved cholera surveillance, accurate outbreak prediction, and targeted interventions [82].

## 8. Recommendations

### 8.1. Recommendations Addressing Socioeconomic Drivers

Environmental surveillance plays a critical role in cholera prevention and outbreak management, yet it remains underutilized in Nigeria. Regular monitoring of water quality and detecting *Vibrio cholerae* in water sources before outbreaks occur could help mitigate large-scale transmission. Deploying portable diagnostic tools, such as biosensors and isothermal amplification devices, in high-risk areas can provide timely and accurate data on water contamination. These technologies are particularly effective in flood-prone regions, where water contamination is a leading cause of cholera outbreaks. Additionally, integrating molecular diagnostics into environmental monitoring programs can enhance the detection of specific *Vibrio cholerae* strains, enabling public health authorities to anticipate antibiotic resistance trends and adapt treatment protocols accordingly. Establishing a standardized framework for environmental surveillance, including routine testing and transparent reporting of water quality data, would allow for proactive interventions such as water purification campaigns and infrastructure improvements in vulnerable communities.

### 8.2. Recommendations on Diagnostics and AI Integration

To effectively manage and mitigate cholera outbreaks in Nigeria, it is imperative to strengthen diagnostic infrastructure nationwide, particularly in rural and underserved regions where outbreaks are most prevalent. The current reliance on clinical suspicion alone for cholera diagnosis often leads to misdiagnosis, as the symptoms overlap with other diarrheal diseases like typhoid and rotavirus infections. Establishing and equipping more microbiology laboratories with advanced diagnostic tools, such as PCR machines and culture facilities, can enhance diagnostic accuracy and provide confirmatory results critical for effective case management. Additionally, scaling up the distribution and use of RDTs like the Crystal VC dipstick test across healthcare centers can ensure faster diagnosis in resource-constrained settings. These tests, which detect *Vibrio cholerae* antigens in stool samples within minutes, are especially useful during outbreaks where time-sensitive interventions can save lives. However, to optimize the use of these diagnostic tools, it is crucial to train healthcare workers on their proper administration and interpretation. National and state health authorities must prioritize continuous training programs to empower health workers with the skills needed to manage diagnostic challenges effectively.

The adoption of AI in cholera outbreak prediction and management presents a significant opportunity to improve Nigeria's public health response. AI-powered predictive models can analyze complex datasets, including environmental, demographic, and epidemiological variables, to forecast high-risk areas for cholera outbreaks. This predictive capability enables more strategic allocation of resources, such as OCVs, water treatment supplies, and healthcare personnel, to regions most likely to experience outbreaks. Additionally, real-time AI applications, such as mobile health platforms, could support healthcare workers in diagnosing and reporting cholera cases quickly, particularly in remote areas. These platforms can also facilitate community-level education programs by providing targeted messaging about hygiene practices, safe water use, and the importance of seeking prompt medical care during outbreaks. By integrating AI tools into its cholera management strategy, Nigeria can enhance its capacity for early detection, rapid response, and effective prevention, ultimately reducing the disease burden and improving health outcomes across the country.

### 8.3. Policy Adjustments and Intervention

The successful implementation of these recommendations requires coordinated efforts among key stakeholders in Nigeria's health sector. At the federal level, the Federal Ministry of Health (FMOH) will take the lead in policy formulation and strategic planning. This includes allocating budgetary resources for laboratory infrastructure upgrades and the procurement of diagnostic tools such as RDT kits and PCR machines. The FMOH will collaborate closely with the NCDC to develop standardized protocols for cholera diagnostics and establish national guidelines for sample collection, testing, and reporting. The NCDC will also coordinate nationwide surveillance activities, ensuring that data collected from diagnostics feeds into a central system for real-time outbreak monitoring and decision-making. These federal bodies will also liaise with international organizations to secure technical expertise and funding for large-scale diagnostic interventions.

At the state level, the State Ministries of Health will be responsible for implementing federal policies and adapting them to meet regional needs. This includes identifying high-priority areas for laboratory establishment or upgrades and ensuring that healthcare facilities in these locations are equipped with RDT kits and basic diagnostic tools. State-level agencies will also oversee the recruitment and training of laboratory technicians, community health workers, and clinicians to enhance their diagnostic capacity. Periodic assessments of laboratory functionality, supply chain efficiency, and healthcare worker competency will be conducted to ensure sustained progress. State health ministries will further work with local governments to strengthen referral systems, ensuring that complex cases identified at primary healthcare centers can be escalated to well-equipped regional laboratories for confirmatory testing. International partners, such as the WHO, UNICEF, and Gavi, the Vaccine Alliance, will play a critical role by providing funding, technical support, and capacity-building initiatives. These partners can assist with the procurement of advanced diagnostic tools and facilitate the training of healthcare workers through workshops and technical courses. Community engagement is equally essential, with local leaders playing a pivotal role in mobilizing their communities for health education campaigns, encouraging early reporting of suspected cases, and fostering the adoption of new diagnostic tools. Communities will also be instrumental in providing feedback on the accessibility and usability of diagnostic services, ensuring that interventions are culturally appropriate and effective.

## 9. Conclusion

This review provides a comprehensive analysis of the cholera outbreaks in Nigeria, tracing the historical patterns, identifying the current drivers, and suggesting the way forward. The findings highlight the persistent nature of cholera as a significant public health challenge in Nigeria, exacerbated by factors such as poor sanitation, inadequate access to clean water, high population density, and economic instability. The study highlights the troubling trend of increasing cholera cases and fatalities in recent years, particularly in densely populated areas like Lagos. It also emphasizes the importance of timely and accurate diagnosis, effective rehydration therapy, and targeted public health interventions, such as vaccination campaigns and improved water and sanitation infrastructure.

Furthermore, the study identifies critical gaps in the current understanding of cholera in Nigeria, including the need for better surveillance of antibiotic resistance, the role of environmental reservoirs, and the influence of socioeconomic and behavioral factors on the spread of the disease. Addressing these gaps through targeted research and the implementation of robust public health strategies is essential for reducing the burden of cholera in Nigeria and achieving long-term control of the disease. The conclusions drawn from this study serve as a call to action for policymakers, healthcare providers, and international organizations to intensify efforts in combating cholera and improving public health outcomes in Nigeria.

## Figures and Tables

**Table 1:** Major cholera outbreaks in Nigeria till date—key figures.

Year	Outbreak origin	Reported cases	Deaths	Case fatality ratio (%)
1970–1971	Near Lagos	22,931	2945	12.8
1991	Kano state	60,000	7654	12.9
1999	Kano state	26,358	2085	7.9
2010–2011	Borno state	64,241	2341	3.8
2018	Kano/Kaduna states	42,466	830	1.95
2021	—	111,062	3604	3.2
2022	—	73,909	2373	3.2
2023	—	57,196	1729	3
2024 (up to epidemiological week 39)	Lagos	10,837	359	3.3

**Table 2:** Comparison of cumulative suspected cases at epidemiological Week 39 in 2023 and 2024.

Year	2023	2024	% increase
Suspected cases	3387	10,837	220
Deaths	106	359	239
Case fatality ratio (%)	3.1	3.3	6

**Table 3:** AI-driven tools and technologies enhancing cholera diagnostics.

Authors	Software/app/product	Summary
Krizhevsky et al.	TensorFlow	AI-powered tools analyzing data from biosensors for colorimetric or fluorescence-based detection of *Vibrio cholerae* DNA or toxins, ensuring automated and accurate results.
Poplin et al.	DeepVariant	Machine learning algorithm for rapid genomic data processing, enabling strain identification and antimicrobial resistance prediction.
Hadfield et al.	Nextstrain	Real-time tracking of *Vibrio cholerae* pathogen evolution using phylogenetic visualization and genomic data integration.
Gootenberg et al.	SHERLOCK	CRISPR-based detection system enhanced by AI to optimize guide RNA, improving sensitivity and specificity for cholera DNA detection.
Gao et al.	AI predictive analytics	Integrates diagnostic data with environmental variables to predict cholera hotspots and guide interventions.
Yan et al.	Multiplex real-time assay	Rapid identification of *Vibrio cholerae* serogroup and toxigenicity using a novel real-time multiplex assay.
Truong et al.	MetaPhlAn2	AI-powered metagenomic tool for analyzing mixed microbial samples, identifying *Vibrio cholerae*, and distinguishing co-infections.
García-Bernalt Diego et al.	SMART-LAMP	Smartphone-operated device for real-time, colorimetric, point-of-care diagnosis via loop-mediated isothermal amplification (LAMP).

**Table 4:** Global AI applications in cholera prediction, surveillance, and management.

Study location	Model type	Key predictors	Performance metrics	Key findings/challenges
Coastal India	Random forest	Chlorophyll-a, sea surface salinity, land surface temperature	Accuracy: 99% F1 score: 0.942 Sensitivity: 0.895	Seasonal drivers are difficult to pinpoint due to covariation among environmental variables.

Tanzania	XGBoost, K-NN	Seasonal weather changes	Balanced accuracy: 0.772 Sensitivity: 0.801 Specificity: 0.742	Oversampling with XGBoost performed best; modeling linked to weather changes highlights variability challenges.

Bangladesh and the USA (cholera-naive population)	ML with antibody profiling	Vibriocidal antibodies, serological markers	cvAUC: 98.2% (naive population) AUC: 91.6% (Bangladesh, 200-day infection window)	Enables high specificity/sensitivity in identifying recent infections; complements clinical efforts.

## Data Availability

Data sharing is not applicable to this article as no new data were created or analyzed in this study.
